# Sexuality in patients treated for borderline personality disorder at the Arrazi psychiatric hospital in Salé

**DOI:** 10.1192/j.eurpsy.2024.374

**Published:** 2024-08-27

**Authors:** N. Ait Bensaid, Y. Bensalah, M. Sabir, F. El Omari

**Affiliations:** psychiatric hospital ararzi, salé, Morocco

## Abstract

**Introduction:**

Borderline personality disorder is a severe mental disorder characterized by generalized instability of emotional regulation of interpersonal relationships and self-image, and marked impulsivity. Several features of this disorder are likely to be associated with problematic sexual health, such as impulsivity (impulsive sexual behavior), identity disorders (unstable sexual identity) and unstable and intense interpersonal relationships. In addition, childhood sexual abuse and violence are common in people’s histories.

**Objectives:**

Assessing sexuality in patients followed for borderline personality disorder at Arrazi Salé psychiatric hospital.

**Methods:**

This is a descriptive cross-sectional study using a questionnaire including socio-demographic criteria with a questionnaire on sexual behavior in female patients followed for borderline personality disorder at the Arrazi Salé psychiatric hospital. Inclusion criteria: women over 18 years of age diagnosed with borderline personality disorder. Exclusion criteria: psychosis, intellectual disability.

**Results:**

We collected 45 patients with borderline personality disorder. The average age was 22, 80% were single, 58% unemployed, 46% had dropped out of high school. The majority of participants were using psychoactive substances. 25% had attempted suicide. 83% were victims of childhood sexual abuse. The majority were significantly more likely to engage in sexual activity at a younger age than their peers. Over 60% had never used contraception 10% had their first pregnancy at a younger age, with termination. 15% have had genital infections. 53% were attracted to both sexes, and over 66% had more than one sexual partner. Over 73% did not experience sexual satisfaction (sexual satisfaction scale less than 10).

**Conclusions:**

The results indicate that sexuality in patients with borderline personality disorder is present early in the course of the disorder, often at a young age, with significant physical, mental and social consequences. Primary care mental health, sexual health and sexual assault services need to be attentive to the clinical diagnosis of this personality disorder, as the nature of the disorder represents both a risk factor and a health threat.

**Disclosure of Interest:**

None Declared

